# Monoradiculopathy-induced abdominal pseudohernia caused by T11-12 soft disc herniation: a case report and literature review

**DOI:** 10.1186/s12891-023-06536-1

**Published:** 2023-05-29

**Authors:** Wan-Jae Cho, Ki-Won Kim, Bo-Hyoung Kim, Ji-Hyun Ryu

**Affiliations:** Department of Orthopedic Surgery, Yeouido St. Mary’s Hospital, College of Medicine, The Catholic University of Korea, 10, 63-ro, Yeongdeungpo-gu, Seoul, 07345 Republic of Korea

**Keywords:** Abdominal pseudohernia, Thoracic disc herniation, Case report, Literature review

## Abstract

**Background:**

An abdominal pseudohernia is a rare clinical entity that consists of an abnormal bulging of the abdominal wall that can resemble a true hernia but does not have an associated underlying fascial or muscle defect. Abdominal pseudohernia is believed to result from denervation of the abdominal muscles in cases of herpes zoster infection, diabetes mellitus, lower thoracic or upper lumbar disc herniation, surgical injuries, and rib fracture. To date, nine cases of abdominal pseudohernia caused by disc herniation at the lower thoracic or upper lumbar levels have been reported.

**Case presentation:**

A 35-year-old man with no underlying disease or traumatic event presented with chief complaints of left flank pain and a protruding left lower abdominal mass that had formed one day earlier. There was no true abdominal hernia on abdominal computed tomography (CT), although CT and magnetic resonance imaging (MRI) showed a herniated soft (non-calcified) disc into the left neural foramen at the T11-12 level. A nonsteroidal anti-inflammatory drug was prescribed for the flank pain, and the patient was followed on a regular basis for six months. Follow-up MRI taken at the last visit showed complete resorption of the herniated disc. Abdominal pseudohernia and flank pain were also completely resolved.

**Conclusion:**

We report a rare case of monoradiculopathy-induced abdominal pseudohernia caused by foraminal soft disc herniation at the T11-12 level. In patients who have an abdominal pseudohernia without herpes zoster infection, diabetes mellitus, or traumatic events, lower thoracic disc herniations should be included in differential diagnosis.

## Background

Abdominal pseudohernia is a rare clinical entity that consists of an abnormal bulging of the abdominal wall that can resemble a true hernia but has no associated underlying fascial or muscle defect [1]. Abdominal pseudohernia is believed to result from denervation of the abdominal muscles in cases of herpes zoster infection [2], diabetes mellitus [3], lower thoracic or upper lumbar disc herniation [4–10], surgical injuries [11], and rib fractures [1].

The components of the lateral abdominal wall include the external oblique muscles, internal oblique muscles, and transversus abdominis muscles [12]. The external oblique muscles are innervated by the T5 ~ 12 spinal nerves, the internal oblique muscles by the T7 ~ L1 spinal nerves, and the transversus abdominis muscles by the T7 ~ 12 spinal nerves [13]. Any lesions that cause dysfunction of or injury to those spinal nerve(s) or their terminal branch(es) can lead to weakness of the abdominal wall muscles and an resultant abdominal bulging, an abdominal pseudohernia [13].

To date, nine reported cases of abdominal pseudohernia have been caused by disc herniations at lower thoracic or upper lumbar levels (Table 1) [4–10]. Because of the much lower incidence of thoracic disc herniations compared with that of cervical or lumbar cases, thoracic disc herniations might not be suspected as a cause of abdominal pseudohernia. Here, we present a rare case of monoradiculopathy-induced abdominal pseudohernia caused by T11-12 soft disc herniation.

**Table 1:** Pseudohernia in disc herniation patient reported in the literature

Author and year	Age	Sex	Underlying disease	Disc herniation level	Disc herniation location	Treatment
Billet et al. (1989) [5]	62	M		L1-2		Discectomy
Bartolomei et al. (1992) [4]	25	M		L2-3	Left foramen	Discectomy
Stetkarova et al. (2007) [10]	50	F		T12-L1	Bilateral paramedian and foramen	Conservative care
Stetkarova et al. (2007) [10]	34	M		T10-11 T11-12	Left Foramen Left paramedian	Conservative care
Laban et al. (2007) [9]	75	M		T12-L1	Left paramedian	Conservative care
Elgueta et al. (2018) [7]	53	M	None	L1-2	Left lateral recess and foramen	Conservative care
Butenschoen et al. (2020) [6]	81		Hypertension, stroke	T11-12	Right paramedian	Hemilaminectomy
Fitzpatrick et al. (2022) [8]	57	M	None	T12-L1	Left extraforaminal	Conservative care
Fitzpatrick et al. (2022) [8]	67	M	None	T9-10	Right paramedian and foraminal	Conservative care

F, female; M, male

## Case presentation

**Fig. 1:**
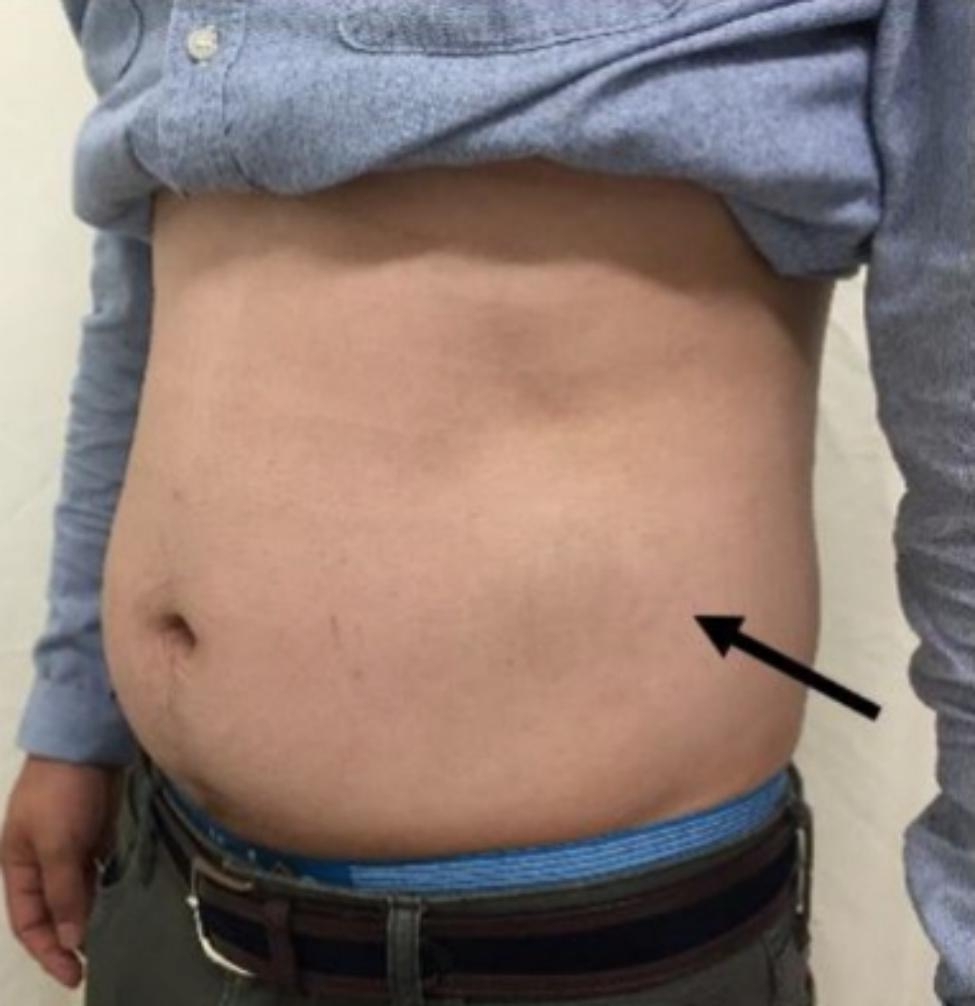
Pseudohernia on the patient’s left trunk (black arrow)

**Fig. 2:**
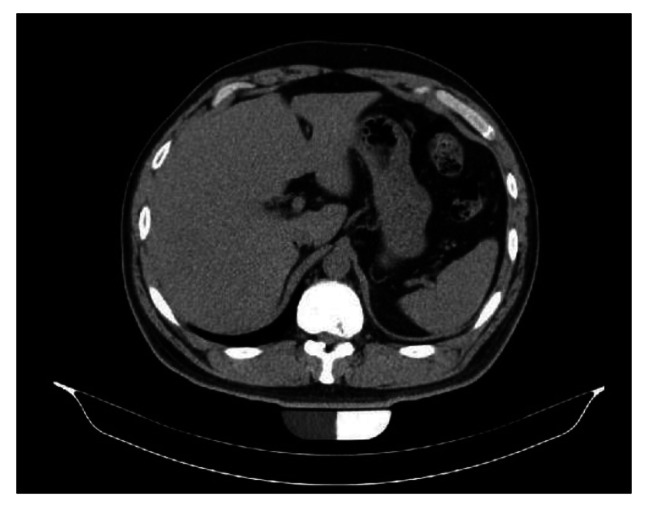
Computed tomography (CT) scan of the abdomen. Axial CT scan showing no true abdominal hernia and no calcification of herniated disc

A 35-year-old man with no underlying diseases or traumatic events was transferred to our orthopedic outpatient clinic with chief complaints of left flank pain and left lower abdominal protruding mass that appeared one day earlier (Fig. 1). Before visiting our outpatient clinic, abdominal computed tomography (CT) had been conducted in the colorectal clinic with suspicion of a true abdominal hernia. However, no such hernia had been found (Fig. 2).

**Fig. 3:**
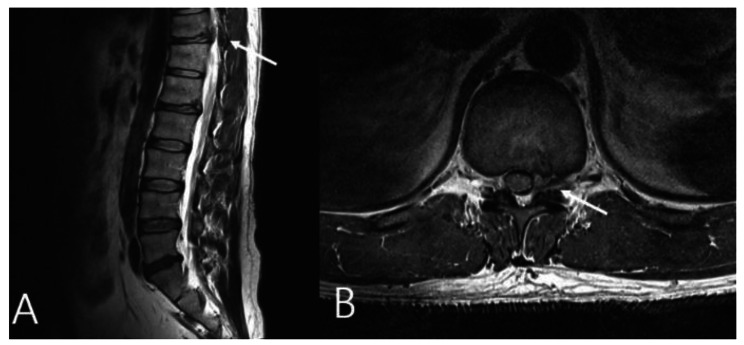
Magnetic resonance imaging (MRI) of the lumbar spine. (**A**) Sagittal T2-weighted MRI showing T11-12 disc herniation (white arrow). (**B**) Axial T2-weighted MRI T11-12 left foraminal disc herniation (white arrow)

**Fig. 4:**
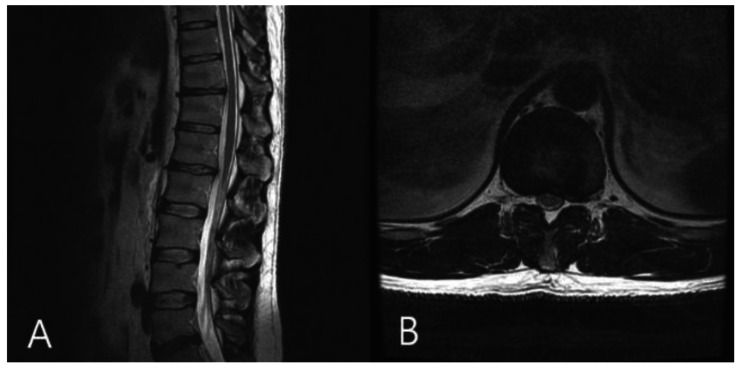
Magnetic resonance imaging (MRI) of the lumbar spine. (**A**) Sagittal T2-weighted MRI showing no abnormal finding. (**B**) Axial T2-weighted MRI T11-12 herniated disc complete resolusion

**Fig. 5:**
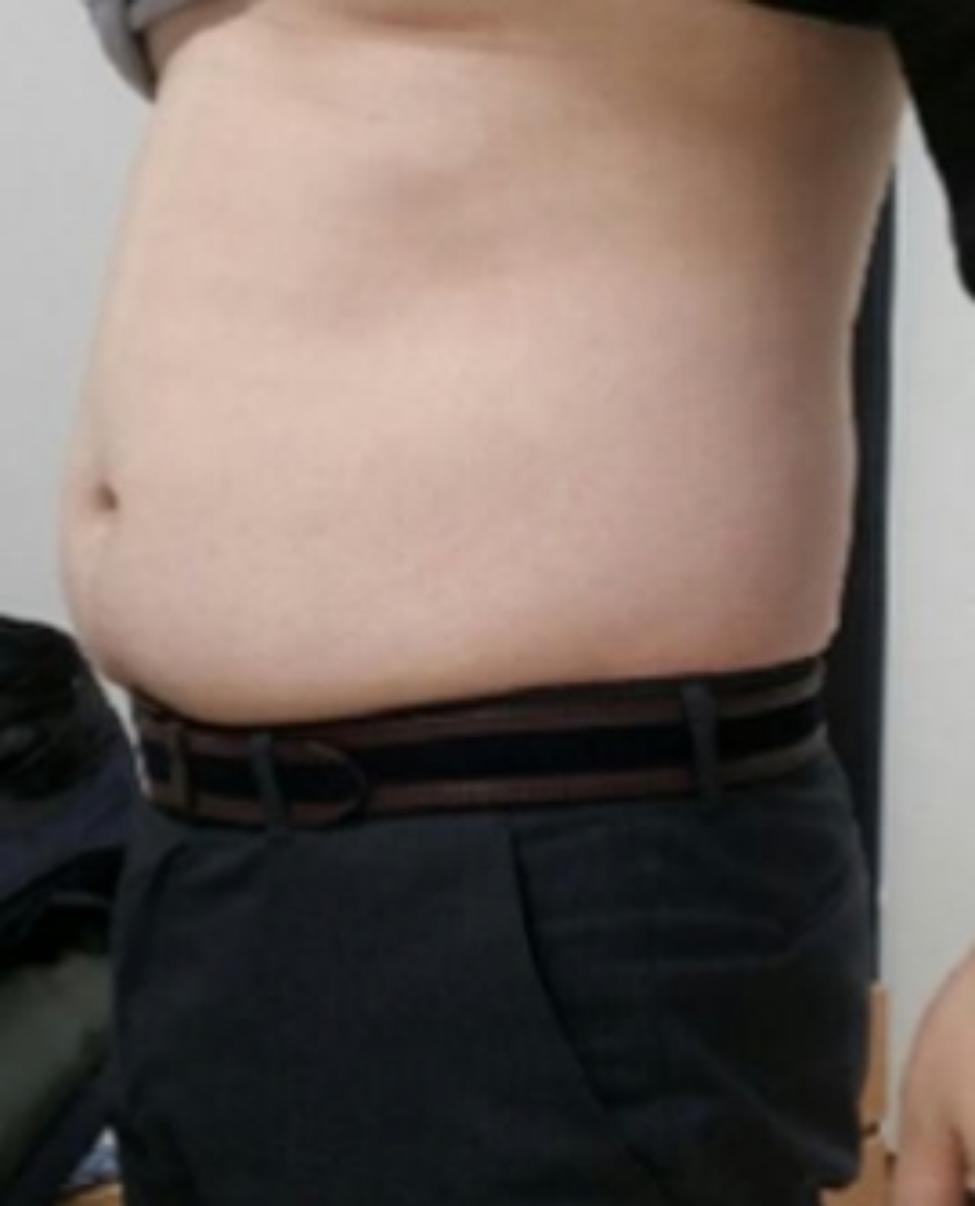
Resolved pseudohernia on the patient’s left trunk

On physical examination, there were no abnormalities in lower extremity muscle strength or sensation, and there were no specific findings except the protruding mass of the left lower abdomen. Plain radiography of the lumbar spine revealed no specific findings. The initial laboratory examinations showed no specific findings. CT and magnetic resonance imaging (MRI) showed a herniated soft (non-calcified) disc protruding into the left neural foramen at the T11-12 level (Figs. 2 and 3). A nonsteroidal anti-inflammatory drug was prescribed for flank pain, and the patient was followed regularly for six months. Follow-up MRI performed at the last visit showed complete resorption of the herniated disc (Fig. 4). Abdominal pseudohernia and flank pain were also completely resolved (Fig. 5).

## Discussion and conclusions

When a patient complains of a protruding abdominal mass, a discrete mass such as cancer and hernia are considered first. After both conditions have been eliminated, pseudohernia may be considered. Abdominal pseudohernia is most often caused by herpes zoster infection and can also result from denervation of abdominal muscles in cases of diabetes, lower thoracic or upper lumbar disc herniation, surgical injury, and rib fracture [1–11]. Therefore, differential diagnosis of the above diseases should be considered in patients with pseudohernia. Abdominal pseudohernia due to thoracic disc herniation is a radiculopathy symptom of abdominal muscle weakness caused by denervated spinal nerves. The incidence of thoracic disc herniation is estimated to be 0.25 to 1% of all disc herniations [14]. Patients with thoracic disc herniation rarely show symptoms, and most of those that do present involve myelopathy [14–16]. Therefore, it is not easy to prioritize thoracic disc herniation in patients who complain of a protruding abdominal mass. In our case, there was no mass-like lesion or hernia observed on CT, skin lesion, underlying disease, or surgical history, and only protruding abdominal mass and flank pain were reported. Considering the possibility of flank pain due to thoracic disc herniation, MRI can be performed. Pseudohernia due to lower thoracic disc herniation can be considered when a patient who does not have a true hernia has abdominal protruding mass without herpes zoster infection, diabetes mellitus, or traumatic event.

To date, nine reported disc herniation patients have developed abdominal pseudohernia [4–10]. The US National Library of Medicine from the National Institutes of Health (PubMed) was used for our literature search. The search used the following major MeSH headings: ‘‘Intervertebral disc herniation’’ and ‘‘Pseudohernia’’. Two authors independently reviewed for relevance. We included only articles published in English and that included human subjects, and excluded paper that did not provide an additional description of the patient. Full texts of the selected papers were reviewed by all two authors and consensus was reached in all cases for inclusion or exclusion. After review there were 7 articles that evaluated intraobserver and interobserver agreement. All nine cases had lesions in the lower thoracic or upper lumbar area. Most were male, the most common treatment was conservative treatment, and the most common level was T12-L1 level. Our case also showed a lower thoracic disc herniation at the T11-12 level and received conservative treatment. Since spinal discs are soft tissue, herniation does not appear as calcification on CT. Conservative treatment is common while waiting for the herniated disc to be resorbed naturally. Although there has been no report of pseudohernia recurrence to date, it is believed that recurrence of disc herniation can cause pseudohernia recurrence, and for this, smoking, diabetes, and disc protrusion, which are known risk factors for recurrent disc herniation, can be considered.

We report a rare case of monoradiculopathy-induced abdominal pseudohernia caused by soft disc herniation at the T11-12 level. In conclusion, if herpes zoster infection, diabetes mellitus, or traumatic event have been eliminated in patients with pseudohernia, evaluation of lower thoracic disc herniation should be considered.

## Data Availability

All data concerning these cases are presented in the manuscript.

## Abbreviations

CT: computed tomography
MRI: Magnetic resonance imaging
